# The Microbiota-Gut-Brain Axis as a Key to Neuropsychiatric Disorders: A Mini Review

**DOI:** 10.3390/jcm10204640

**Published:** 2021-10-10

**Authors:** Katarzyna Stopińska, Maria Radziwoń-Zaleska, Izabela Domitrz

**Affiliations:** 1 Department of Neurology, Faculty of Medical Sciences, Medical University of Warsaw, 01-809 Warsaw, Poland; idomitrz@wum.edu.pl; 2 Department of Psychiatry, Faculty of Medicine, Medical University of Warsaw, 00-685 Warsaw, Poland; mariar@wum.edu.pl

**Keywords:** gut microbiota, neurological disorders, psychiatric disorders

## Abstract

The central nervous system (CNS) is closely related to the gastrointestinal tract, mainly through regulating its function and homeostasis. Simultaneously, the gut flora affects the CNS and plays an essential role in the pathogenesis of neurologic and neuropsychological disorders such as Parkinson’s and Alzheimer’s disease, multiple sclerosis, amyotrophic lateral sclerosis or autism spectrum disorder. The population of gut microorganisms contains more than one billion bacteria. The most common are six phyla: Proteobacteria, Actinomyces, Verucomicrobia, Fusobacteria, and dominant Bacteroides with Firmicutes. The microbiota–gut–brain axis is a bidirectional nervous, endocrine, and immune communication between these two organs. They are connected through a variety of pathways, including the vagus nerve, the immune system, microbial metabolites such as short-chain fatty acids (SCFAs), the enteric nervous system, and hormones. Age, diet, antibiotics influence the balance of gut microorganisms and probably lead to the development of neurodegenerative disorders. In this article, a review is presented and discussed, with a specific focus on the changes of gut microbiota, gut–brain axis, related disorders, and the factors that influence gut imbalance.

## 1. Introduction

In the human body, the number of microorganisms inhabiting the gastrointestinal tract has been estimated to exceed 10^14^ [1]. Bacteria colonize the gut after birth and remain for the rest of life. The gastrointestinal tract of a newborn is sterile. Several minutes are enough for the intestines to be colonized by the mother’s bacteria. The composition of the gut microbiota at this stage depends on the way of giving birth. In the case of vaginal delivery, colonization is faster and different types of bacteria (*Lactobacillus, Prevotella*, and *Sneathia)* [1] overweight when compared to a cesarean section (*Staphylococcus, Corynebacterium*, and *Propionibacterium)* [2]. The composition of gut microbiota is changing in the next few days and during the entire life. From the age of 1 year, the bowel composition is similar to that of an adult. The healthy human gut comprises three groups of bacteria: symbionts, commensals, and pathobionts. The first two are responsible for healthy intestinal microflora. On the other hand, the last group of bacteria can trigger a harmful impact on the host, especially when its superiority is significant. Dietary habits, antibiotics, and probiotics/prebiotics intervention may modify the composition of the gut microbiota and, as a result, affect the functions not only of the gut but also the whole central nervous system.

The collection of bacteria, archaea, and eukarya colonizing the gastrointestinal tract is termed the gut microbiota (GM). The GM is composed of four main phyla (*Bacteroidetes*, *Firmicutes*, *Proteobacteria*, and *Actinobacteria*) and two minor phyla (*Verrucomicrobia* and *Fusobacteria*) [3]. These commensal bacteria interact with one another and the host intestinal epithelium and contribute to intestinal homeostasis and host immunity. Therefore, microbes not only have a positive influence on the health of the host but may also contribute to the development of several diseases, including neurological and mental disorders, such as Parkinson’s disease (PD), Alzheimer’s disease (AD), multiple sclerosis (MS), amyotrophic lateral sclerosis (ALS), and autism spectrum disorder (ASD).

**Figure 1:**
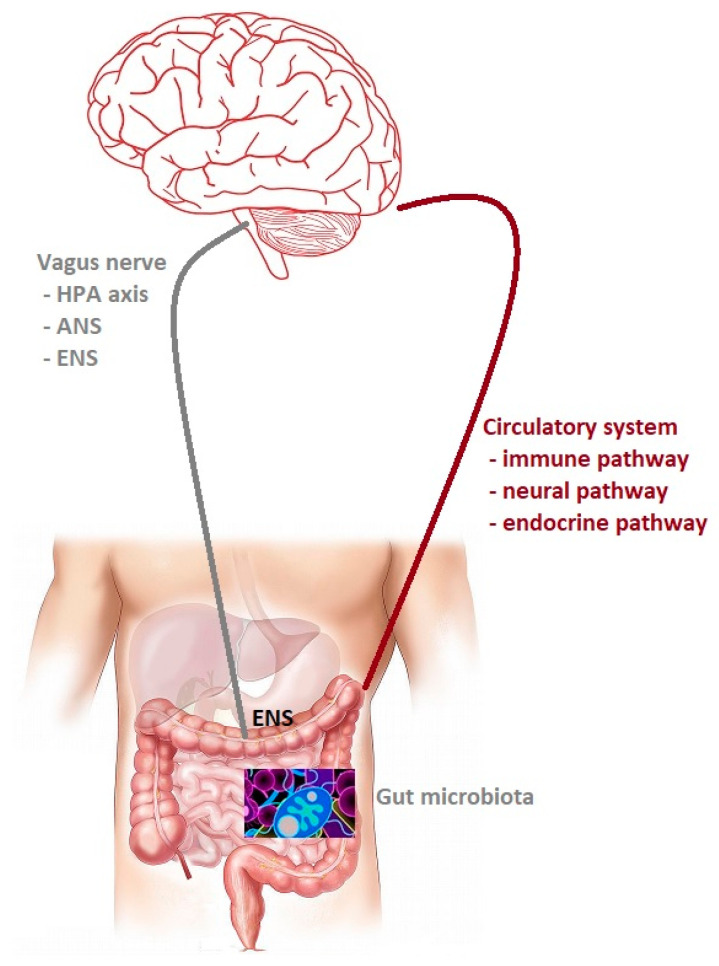
The gut microbiota–brain axis. Bidirectional communication between the gut microbiota and the central nervous system (CNS). The routes of communication involve the autonomic nervous system (ANS), the enteric nervous system (ENS) and the vagus nerve, the neuroendocrine system, the hypothalamic– pituitary–adrenal (HPA) axis, and the immune pathway.

Anatomically, the gut and the central nervous system are involved in a bidirectional communication system with significant implications in health and disease between them, which is termed the gut–brain axis (Figure 1). Initially, the research was concentrated on the influence of the nervous system on the functioning of the digestive system. As a result, it is known that gut microbiota can modulate connection of them to maintain homeostasis. This crosstalk consists of multiple pathways, including the autonomic nervous system, the enteric nervous system (ENS), and hypothalamic-pituitary-adrenal, which use the vagus nerve for communication and immune, endocrine, and neural pathways use circulation for that [4]. The enteric nervous system is a quasi-autonomous part of the nervous system, which consists of two ganglia: the myenteric (Auerbach’s) and submucosal (Meissner’s) plexus, which together modulate the digestive system. Myenteric plexuses are located between the inner and outer layers of the muscularis externa, while submucosal plexuses are located in the submucosa. The ENS is a web of sensory neurons, motor neurons, and interneurons of the autonomic nervous system. It is responsible for gut function such as digestion, gut motility and permeability secretion of bile, carbohydrate levels, mechanical distortion of the mucosa, maintenance of epithelial fluid level, luminal osmolality, mucus production, and mucosal immune response [5]. Not only ENS communicates with the central nervous system; there is growing evidence that gut microbiota can also do it, using their metabolites and neurotransmitters with neuromodulatory properties, such a tryptophan, 5-hydroxytryptamine (5-HT), gamma-aminobutyric acid (GABA), glutamine, histamine, short chain fatty acids (SCAFs), catecholamines and many others [4]. Serotonin (5-hydroxytryptamine, 5-HT), a neurotransmitter, is released from enterochromaffin (EC) cells in response to a number of stimuli, including signals from the gut microbiota. 5-HT is produced by microbes with the aid of the enzyme tryptophan hydroxylase 1 (TPH1). The gut microbiota can regulate host tryptophan levels by influencing its metabolism. Several bacteria belonging to *Lactococcus*, *Lactobacillus*, *Streptococcus*, *Escherichia coli*, and *Klebsiella* have been reported to be able to produce serotonin by expressing tryptophan synthetase. Based on the evidence in animal models, luminal tryptophan can be metabolized by the other gut microbiota, limiting its availability for the host [6]. GABA, an inhibitory neurotransmitter, can also be produced by host/microbes from amino acid glutamate. *Escherichia* spp. and *Lactobacillus* spp. can synthesize it. *Lactobacillus* and *Bifidobacterium* increase the level of GABA in ENS. Glutamine is produced by bacteria such as *Corynebacterium glutamicum*, *Brevibacterium* spp., *L. plantarum*, and *L. lact* is or absorbing from dietary [7]. The glutamate plays a vital role in the pathophysiological changes of neuronal excitability.

Short chain fatty acids, particularly acetic, propionic, and butyric acid, are the main end product of bacterial fiber fermentation in the gut [8]. They are produced by many types of families such as *Prevotellaceae*, Bacteroides, and Firmicutes. Studies have shown that SCFAs directly affect the permeability of the blood–brain and the blood–gut barriers [9] and contain anti-inflammatory and antioxidant properties. SCFAs have been suggested as essential mediators.

The gut microbiota promote the production of several neurotransmitters, peptides, and short-chain fatty acids, as well as regulatory T and B cells. In that way, GM redounded to maintain gut permeability, decreases lipopolysaccharides to the periphery, reduces blood–brain barrier disruption, and finally activates brain immune and neural cells.

The gut microbiota play a crucial role in brain development, behavior, and host immune system. The understanding of the trajectory of neurological diseases requires a focus on gut bacteria and communication between them and the CNS. The increased intestinal permeability contributes to the misplacement of some types of bacteria, their neuroactive metabolites and finally induces neuroinflammation. The aim of this article is a review and summary of the knowledge on the changes of gut microbiota, gut–brain axis, related disorders, and the factors which influence gut imbalance.

Particular neurological diseases such as PD, AD, MS, or ALS have their own different composition of gut microbiota. Dysbiosis, resulting in the decline of neurotransmitters, short-chain fatty acids, or low intestinal permeability, can trigger the onset of a disorder.

## 2. Parkinson’s Disease

Parkinson’s disease (PD) is the second most common neurodegenerative disorder after Alzheimer’s disease. It is a neurodegenerative movement disorder with onset in the sixth decade of life. The main hallmark involved in pathology is alfa-synuclein (alfa-syn), a protein that abnormally accumulates and aggregates in the form of Lewy bodies and Lewy neurites [10]. Moreover, PD is characterized by the loss of dopaminergic neuronal cells in the substantia nigra (SN) pars compacta that project to the striatum [11]. Next to classic motor symptoms of PD, including bradykinesia, resting tremor, muscular rigidity, and postural instability, there are many non-motor symptoms such as autonomic dysfunction, olfactory impairment, cognitive or mood deficits, and sleep disturbances. Most of them appear a few years before the motor symptoms and are attributed to the sequential development of Lewy bodies in various brain regions. According to the Braak hypothesis, the olfactory bulb, the dorsal motor nucleus of the vagal nerve, locus coeruleus, raphe nucleus, basal nucleus of Meynert, and pedunculopontine nucleus are affected first [12]. These authors suggested a possible starting point and downstream pathway of PD pathology. The damage extends via the olfactory tract or the dorsal motor nucleus of the vagus nerve (DMVN), and alfa-syn inclusions spread in other specific brain areas, ultimately occupying substantia nigra and cortex [12,13]. Based on the research models carried out with the use of the alfa-syn preformed fibril (PFF) directly into the muscular layers of pylorus and duodenum, it is known that it started alfa-syn aggregation and degeneration of dopaminergic neurons [14]. In addition, it shows that the gut–brain axis is involved in the transmission of pathologic alfa-syn, which was concluded from the research on vagotomy before and after injection [15]. Moreover, Braak et al. [13] have found that alfa-syn pathology starts in the Meissner’s plexus of the enteric nervous system (ENS) and via vagal preganglionic axons using retrograde axon transport achieves DMVN in the medulla oblongata. Based on this hypothesis, Holmqvist et al. [16] have provided the first experimental evidence by using different types of alfa-synuclein forms in rats and demonstrate that they are transported from the gut to the brain via the vagal nerve. This gave a beginning to numerous studies [17,18], which show that alfa-syn can translocate between CNS and ENS, and truncal vagotomy could be a prevention method before illness [19,20,21]. Notably, it seems that the gastrointestinal tract contains alfa-syn not evenly but with a falling rostrocaudal gradient. The upper parts of the digestive system (submandibular gland or esophagus) have the largest amount of protein compared to the lowest parts [22], which coincides with the distribution of vagal innervation [23].

### Bacterial Products-Short-Chain Fatty Acids (SCFSs) in PD

In the study assessment, fecal SCFAs levels in PD patients and control subjects, particularly butyric acid, were linked to microbiota changes. Reduction of SCFA production by bacteria leads to increased colonic inflammation, gut leakiness, and increased risk of alfa-syn depositions in the gastrointestinal tract [24,25]. Butyric acid is also able to regulate some genes related to brain plasticity and regeneration. Worth mentioning is that sodium butyrate is used in treatment to prevent the MPTP-induced dopaminergic neurodegeneration process in an animal model. Furthermore, it repairs the DNA damage induced by alfa-syn or integrates the blood–brain barrier or the gut barrier.

In many different studies, which are based on fecal or mucosal samples collected from PD patients and control samples, it is known that microbial taxa have changed even at an early stage of the disease. Notably, the last meta-analysis of 15 case–control studies across different geographical regions in PD population has shown very interesting findings, i.e., a decrease in *Prevotellaceae*, *Faecalibacterium*, and *Lachnospiraceae*, as well as an increase in *Bifidobacteriaceae*, *Ruminococcaceae*, *Verrucomicrobiaceae*, and *Christensenellaceae* [26]. These changes of gut microbiota have an effect on symptoms. The low level of Prevotella and a higher level of Bifidobacterium and Lactobacillus are clearly associated with a reduction of ghrelin. The function of ghrelin is to participate in regulating dopaminergic neuron function in the substantia nigra pars compacta. In this way, its acylated isoform is used in an MPTP-induced mouse model of PD as a neuroprotective effect in dopaminergic neurons of SN. The concentration of ghrelin is low in PD population regardless of the stage [27,28,29,30]. Prevotella is associated with thiamine biosynthesis and *Bacteroides* with riboflavin biosynthesis. A low level of thiamine is connected with olfactory dysfunction in an early stage of PD [31]. The abundance of Prevotellaceae decreased with the development of the disease [32]. Therefore, the level of this type of bacteria is considered as an illness biomarker. The family *Faecalibacterium* performs a similar function in producing SCFAs and anti-inflammatory metabolites. A similar decline can be observed with the progress of PD [33]. On the other side, even though a Bifidobacteriaceae family regulates immunity or inhibits the growth of harmful gut bacteria, seven studies showed similar results, indicating that the population of this family of bacteria was increased in PD patients. In many studies, the same results have been observed. Opportunistic pathogens and carbohydrate-metabolizing probiotics are higher than average. Finally, short-chain fatty acids (SCFAs) producing bacteria are reduced in PD [34].

## 3. Alzheimer’s Disease

Alzheimer’s disease (AD) is the most common neurodegenerative disorder, where dementia symptoms gradually worsen over several years. There are over 50 million people worldwide living with dementia in 2020. It is estimated that this number probably will double every 20 years, reaching 82 million in 2030 and 152 million in 2050 [35]. The basis of the disease is a progressive loss of cholinergic function, as well as neuronal cell death in the hippocampus and cerebral cortex. There are two types of AD. The most common (around 95% of cases) is sporadic AD and less than 5% suffer from familiar AD. The latter is caused by monogenic mutations in the genes for amyloid precursor protein (APP) and both presenilin genes (PRES 1 and PRES 2) [36]. The carriers of this mutation will definitely fall ill with AD. Alzheimer’s disease pathology is characterized by two specific neuropathological features: extracellular amyloid plaques and intracellular neurofibrillary tangles [37]. The above-mentioned specific features allow for the diagnosis of Alzheimer′s disease, but the symptoms of the disease mainly relate to extensive loss of synapses or marked loss of neurons later in the disease [36,38]. The presence of pathological proteins contributes to the activation of glial cells in the brain, triggering inflammatory processes such as the release of free radicals, excitatory amino acids, inflammatory interleukins and nitric oxide. These substances contribute to the death of neurons and their connections [39]. Gut microbiota may modulate host brain function and behavior via the microbiota–gut–brain axis, including cognitive behavior.

Glutamate, the excitatory neurotransmitter, and *n*-methyl-D-aspartate (NMDA) receptor play a role in the pathophysiology of AD. NMDA receptors are responsible for learning and memory. The relationship between gut microbiota and NMDA receptor expression was observed. The level of NMDA receptors is significantly reduced after antibiotics administration in a mouse model [40,41]. Cyanobacteria, also known as blue-green algae, can produce neurotoxin β-*n*-methylamino-L-alanine (BMAA) and can be incorrectly inserted into brain proteins leading to misfolding. Chronic dietary exposure to the cyanobacterial toxin can trigger the development of neurofibrillary tangles and Aβ deposits in the brain and increases the risk of AD [42,43]. Other types of cyanobacteria produce neurotoxins such as saxitoxin and anatoxin-α, which can contribute to the process of aging [44].

Some types of bacteria take part in the production of many neurotransmitters. *Lactobacillus brevis* and *Bifidobacterium dentium* can produce γ-aminobutyric acid (GABA). Reduction of these two phyla in the diet will influence the production of GABA in the gut and then lead to a decrease of GABA in CNS [45]. Gut microbiota play an important role in the synthesis of serotonin. The concentration of 5-TH was around 60% lower than model mouse with normal gut microbiota [46].

Brain-derived neurotrophic factor (BDNF) is a protein synthesized in the brain and distributed to different regions of CNS. This protein is involved in several processes. The level of BDNF is decreased not only in the brain but also in the serum. Notably, that gut microbiota may affect host cognition by regulating the expression of BDNF and eventually induce AD [45]. Gut microbiota are also responsible for the production of all kinds of vitamins, including vitamin B12. Low levels of that vitamin in serum are associated with an increased risk of AD and mild cognitive impairment (MCI) [47].

There are also other possible mechanisms of pathogenesis of AD. The first is due to an increase of the *Firmucutes/Bacteroides* ratio causing APP accumulation in the gut. Based on a mouse model APP/PS1 and dysbiosis, an increase of Aβ deposition in the brain and the myenteric neurons in the early stages of illness has been observed. Another animal model indicates that Aβ deposition firstly begins in the gut. A confirmation of this fact has been provided by autopsies of AD patients and the presence of Aβ deposits in their intestines [48].

A second possible mechanism are bacterial metabolites, such as trimethylamine *n*-oxide (TMAO), bile acids (BAs), SCAFs, and hydrogen (H_2_). TMAO is synthesized by host organisms from carnitine, betaine, and choline. This metabolite increases activation of β-secretase, leading to Aβ accumulation, by using host platelets to reach the brain. Bile acids, which are produced by some types of bacteria, can increase blood–brain barrier (BBB) permeability, thus facilitating the accumulation of cholesterol in the brain and further increase of Aβ production. Summarising, dysbiosis increases the amount of TMAO and amyloids while simultaneously decreasing a number of beneficial factors such as hydrogen or SCFAs, which in turn contributes to AD pathology [47].

Finally, it is worth mentioning that Gram-negative bacteria producing lipopolysaccharide (LPS) lead to neuroinflammation processes. The predominance of pro-inflammatory bacteria (*Schigella*/*Escherichia*) over anti-inflammatory is associated with cognitive impairment and amyloidosis in the brain [48].

## 4. Multiple Sclerosis

Multiple sclerosis (MS) is a chronic autoimmune-mediated neurological disease of the central nervous system, which is the first cause of disability in young patients. The exact causes are still unknown. It is believed, however, that genetically predisposed people who are exposed to adverse environmental factors are at risk of developing MS. Based on a systematic review and literature analysis, none of the studies showed any significant differences in the overall gut microbiota composition in MS group compared to controls [49,50]. Only two studies showed increased numbers of *Akkermansia* and *Metanobrevibacter* and a decrease of *Prevotella* [51], *Bacteroides* (coprophilus and fragilis), and *Faecalibacterium prausnitzii* in the MS population relative to controls [49]. The multiple sclerosis model of experimental autoimmune encephalomyelitis (EAE) has suggested a potential role of SCFAs in development and progression in mice, but it has not been confirmed in humans. Furthermore, a higher incidence of brain autoimmunity was observed after transplantation to transgenic mice from MS patients when compared with healthy individuals [52].

## 5. Amyotrophic Lateral Sclerosis

Amyotrophic lateral sclerosis (ALS) is a neurodegenerative, neuromuscular disease characterized by progressive loss of motor neurons. Typically, death due to respiratory paralysis occurs in 3 to 5 years. Degeneration of motor neurons is accompanied by neuroinflammatory processes, with a proliferation of astroglia, microglia, and oligodendroglial cells [53,54]. In some studies, it was observed that transgenic mice, even at the presymptomatic stage, show evidence of dysbiosis. Among a few types of different bacteria which can be related to the pathogenesis of ALS *Akkermansia muciniphila* should be highlighted [54,55].

## 6. Autism Spectrum Disorder

In the last 50 years, the definition of autism spectrum disorders evolved from a rare and quite narrow to widespread although heterogeneous disorder. The basic features of ASD are, first of all, deficits in social communication and repetitive and unusual sensorimotor behaviors. In addition, most patients experience comorbid gastrointestinal disturbances such as abdominal pain, flatulence, constipation, or diarrhea, which indicates the participation of the gut microbiota. In most studies, the composition of intestinal microflora is different in people with psychiatric disorders and healthy individuals. The predominance of *Bifidobacterium* and the reduced amount of *Roseburia* and *Faecalibacterium* in patients with mental disorders is noticeable. As a result, the gut bacteria responsible for producing short-chain fatty acids are likely to be less numerous in people with mental disorders [56].

## 7. Discussion and Conclusions 

The etiology of many nervous system diseases is multifactorial and still not fully understood. Among known genetic factors (PARK genes for PD or PSEN1 and PSEN2 for AD), numerous environmental factors, such as gut microbiota disturbances, are also taken into account. The role of the gut microflora in the proper functioning of humans has been known for a long time. In recent years, its influence on the development of neurological diseases is the subject of extensive research efforts. With the aging of the population and the increasing incidence of neurodegenerative diseases such as Alzheimer’s disease, Parkinson’s disease, multiple sclerosis, or amyotrophic lateral sclerosis, the obtained research results lead us to identifying possible causes of a particular disease. The same conclusion is drawn in the case of psychiatric illnesses such as ASD. Over 2000 years ago, the Ancient Greek physician Hippocrates suggested that “all diseases begin in the gut”. It is hard to disagree with this statement, considering the Braak hypothesis or bidirectional communication between these two organs. The intestinal microbiota affect the homeostasis of the entire system by participating in the production of specific metabolites that regulate and support not only the digestive system but also other systems, especially the nervous and immune systems.

In the case of both quantitative and qualitative changes in the microbiota have occurred. Highly specific microbiota disturbances for various disorders are listed in Table 1. The neurotransmitters and metabolites produced by different bacteria species are presented in Table 2. 

So far, it is difficult to determine whether these changes are primary or secondary to the ongoing disease. Furthermore, each type of bacteria is associated with the production of different neurotransmitters or their metabolites. The elimination or significant predominance of certain types of bacteria may reduce the production of their metabolites, thus contributing to the development of diseases. The changes in gut microbiota can be indicated as a potential biomarker of developing neurological disorders. Therefore, it seems interesting to use these changes to determine treatment in the future. As described so far, intestinal dysbiosis observed in various chronic inflammatory disorders, such as recurrent Clostridium difficile infections, is being treated using fecal microbiota transplantation (FMT) from a healthy population. Changes in the microflora may be two-fold. They may be one of the causes of the disease, but also a symptom, often at an early stage of development.

## Figures and Tables

**Table 1:** Relation between altered gut microbiota composition and particular disorder of the nervous system (↑ = increased, ↓ = decreased).

Disease	Altered Gut Microbiota
Alzheimer’s disease	Bacteroides↑, Tenericutes↑, Firmicutes↓, Verrucomicrobia↓, Proteobacteria↓, Actinobacteria↓, Allobaculum↓, Akermansia↓
	Bacteroides↑, Firmicutes↓, Bifidobacterium↓
Parkinson’s disease	Enterobacteriaceae↑, Prevotellaceae↓
Sclerosis multiplex	Akkermansia↑, Metanobrevibacter↑, Prevotella↓, Bacteroides↓ Faecalibacterium prausnitzii↓
Amyotrophic lateral sclerosis	Akkermansia muciniphila↓
Autism spectrum disorder	Bifidobacterium↑, Roseburia↓, Faecalibacterium↓

**Table 2:** Neurotransmitters/metabolites produced by different type of gut microbiota.

Metabolites	Gut Microbiota	References
GABA	Lactobacillus, Bifidobacterium	[57]
Glutamate	Lactobacillus, Campylobacter jejuni, Coryneform, Bacteroides vulgatus	[58]
Serotonin	Akkermansia muciniphila	[59]
Dopamine	Lactobacillus, Escherichia, Streptococcus, Lactococcus, Bacillus	[58]
Acetylocholine	Lactobacillus, Bacillus	[58]
SCFA	Faecalibacterium, Bifidobacterium longum, Clostridium symbiosum, Lactobacillus fermentum	[58]
